# Supplementary material for: Multiscale modelling of chromatin 4D organization in SARS-CoV-2 infected cells
Source: Nat Commun. 2024 May 13;15:4014. doi: 10.1038/s41467-024-48370-6 (PMC11091192; doi:10.1038/s41467-024-48370-6)
Supplement: Supplementary file 1 — Supplementary Information [file 41467_2024_48370_MOESM1_ESM.pdf]

Supplementary Information

Multiscale modelling of chromatin 4D organization in SARS-CoV-2 infected cells

Andrea M. Chiariello[1,a,b], Alex Abraham[1,a], Simona Bianco[1], Andrea Esposito[1], Andrea Fontana[1], Francesca Vercellone[1], Mattia Conte[1] and Mario Nicodemi[1,2,b]

[1] *Dipartimento di Fisica, Università degli Studi di Napoli Federico II, and INFN Napoli, Complesso Universitario di Monte Sant'Angelo, 80126 Naples, Italy.*
[2] *Berlin Institute for Medical Systems Biology at the Max Delbruck Center for Molecular Medicine in the Helmholtz Association, Berlin, Germany.*

[a] *These authors contributed equally*
[b] *Corresponding authors: andreamaria.chiariello@na.infn.it mario.nicodemi@na.infn.it*

Supplementary Figures

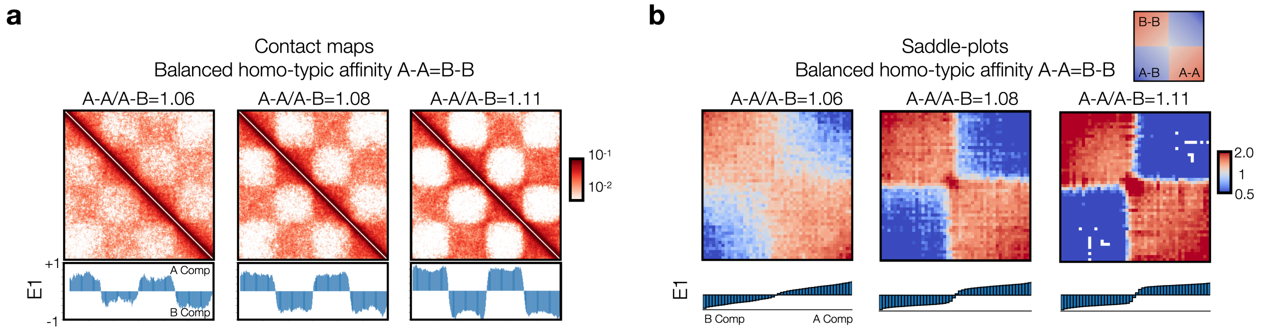

Supplementary Fig. 1: Modelling of chromatin re-structuring in A/B compartments with balanced homo-typic affinities. a Contact maps from MD simulations showing different levels of compartmentalization by variation of balanced (i.e. A-A=B-B) homo-typic interaction. Interaction affinities are normalized with respect the hetero-typic A-B affinity (Methods), kept constant. Below the 1st eigenvector E1 from the PCA is shown. **b** Saddle-plots, computed from models shown in panel a, highlight the enhanced A-B mixing upon decrease of balanced homo-typic interactions. Below, aggregated 1st eigenvector E1, sorted in ascending order, is shown. Source data are provided as a Source Data file.

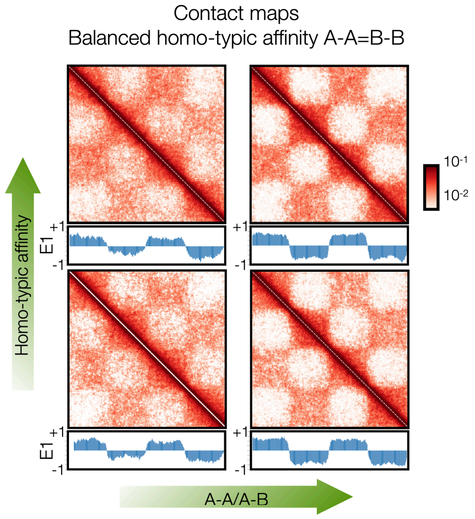

Supplementary Fig. 2: Modelling of chromatin re-structuring in A/B compartments with variable hetero-typic affinity. Increase of hetero-typic A-B binding affinity leads to enhancement of A/B compartment mixing. Heatmaps are computed from a populations of 3D structures obtained from MD simulations. As before, relative affinity A-A/A-B is shown.

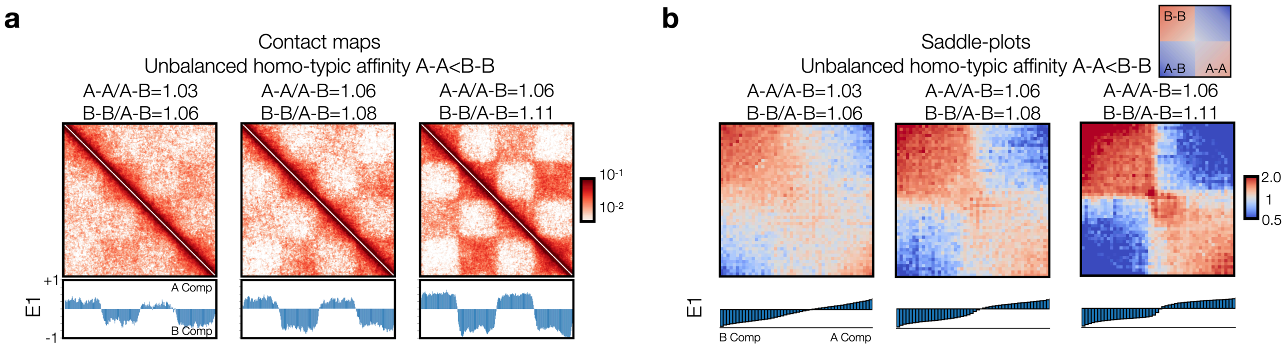

Supplementary Fig. 3: Modelling of chromatin re-structuring in A/B compartments with unbalanced homo-typic affinities. **a** Contact maps from MD simulations showing different levels of compartmentalization by variation of unbalanced (i.e. A-A<B-B) homotypic interaction. Note that decrease of A-A produces both A-compartment weakening and A-B mixing. Interaction affinities are normalized with respect the hetero-typic A-B affinity (Methods), kept constant. Below the 1st eigenvector E1 from the PCA is shown. **b** Saddle-plots, computed from models shown in panel a, highlight the enhanced A-B mixing upon decrease of homo-typic interactions. Below, aggregated 1st eigenvector E1, sorted in ascending order, is shown. Source data are provided as a Source Data file.

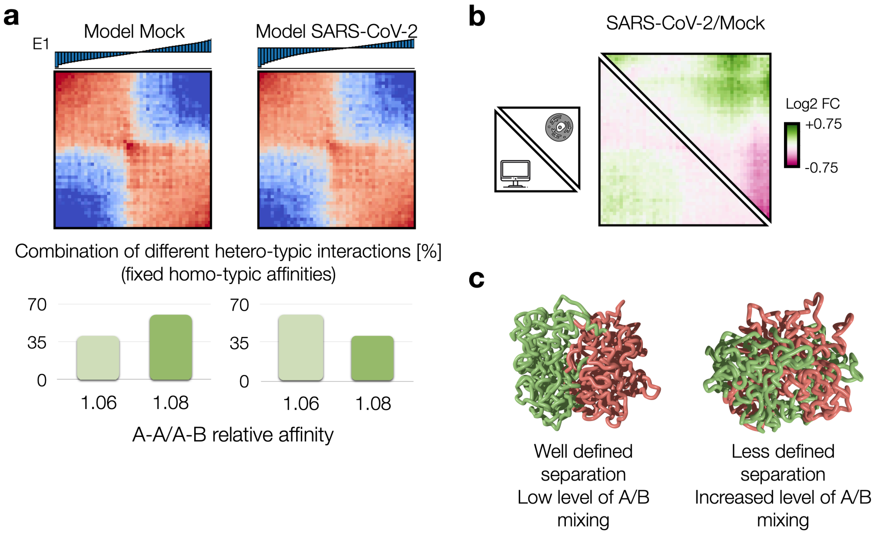

Supplementary Fig. 4: Modelling of chromatin re-structuring in A/B compartments upon SARS-CoV-2 infection combining different hetero-typic affinities. **a** Saddle-plot of best fit polymer model for Mock and SARS-CoV-2 conditions. Above, the sorted 1st eigenvector E1 is shown. Below, best fit coefficients with experimental saddle-plots. Dark and light green bars indicate homo-typic A-A and B-B affinity used in the fit (only balanced models were considered), normalized with respect the background hetero-typic A-B affinity (Methods). **b** Log2 Fold Change of the saddle-plots from the model (bottom left) and HiC data[1] (top right). Pearson correlation between matrices is r=0.6. **c** 3D structures obtained from MD simulations performed with lower (left) and higher (right) A-B affinity. Source data are provided as a Source Data file.

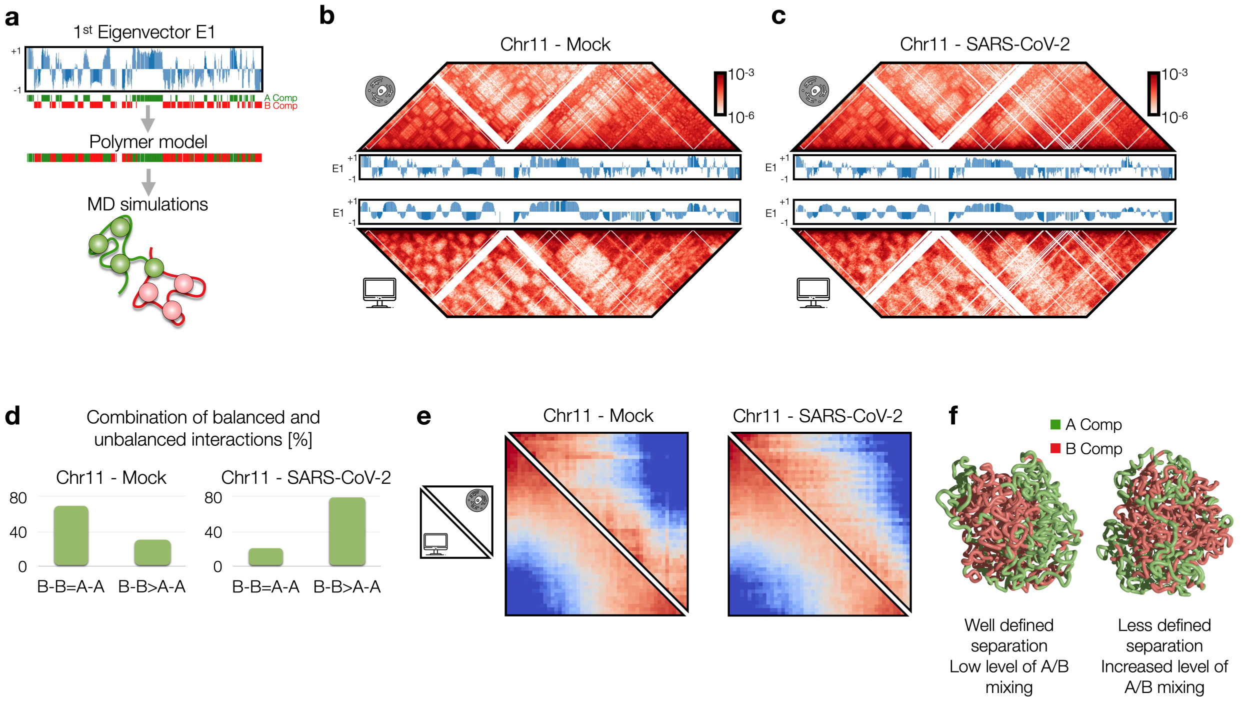

Supplementary Fig. 5: Polymer model including A/B compartment profile reproduces genome organization in Mock and SARS-CoV-2 conditions. **a** We defined a polymer model based on 1st eigenvector of the PCA, from which A/B compartments are defined (Methods). **b** Simulated contact maps in Mock condition accurately recapitulates experimental HiC data (Pearson r=0.94, distance corrected r'=0.5). In the middle, 1st eigenvector of the PCA from HiC (up) and model (bottom) contact maps (Pearson r=0.89). **c** As panel b, for SARS-CoV-2 infected condition (Pearson r=0.9, distance corrected r'=0.5, between HiC and model contact maps, Pearson r=0.89 between experimental and model 1st eigenvectors). **d** Best fit combination of models with balanced and unbalanced homo-typic affinities ($E_{A\text{-}A}/E_{A\text{-}B}$ in the range 1.06-1.08, $E_{B\text{-}B}/E_{A\text{-}B}$=1.08) in Mock and SARS-CoV-2 infected condition. **e** Saddle-plots from HiC data and model of chromosome 11 in Mock (Pearson correlation r=0.92) and SARS-CoV-2 infected condition (Pearson correlation r=0.90). **f** 3D rendering of best polymer models of chr11 in Mock (left) and SARS-CoV-2 (right) infected conditions. Source data are provided as a Source Data file.

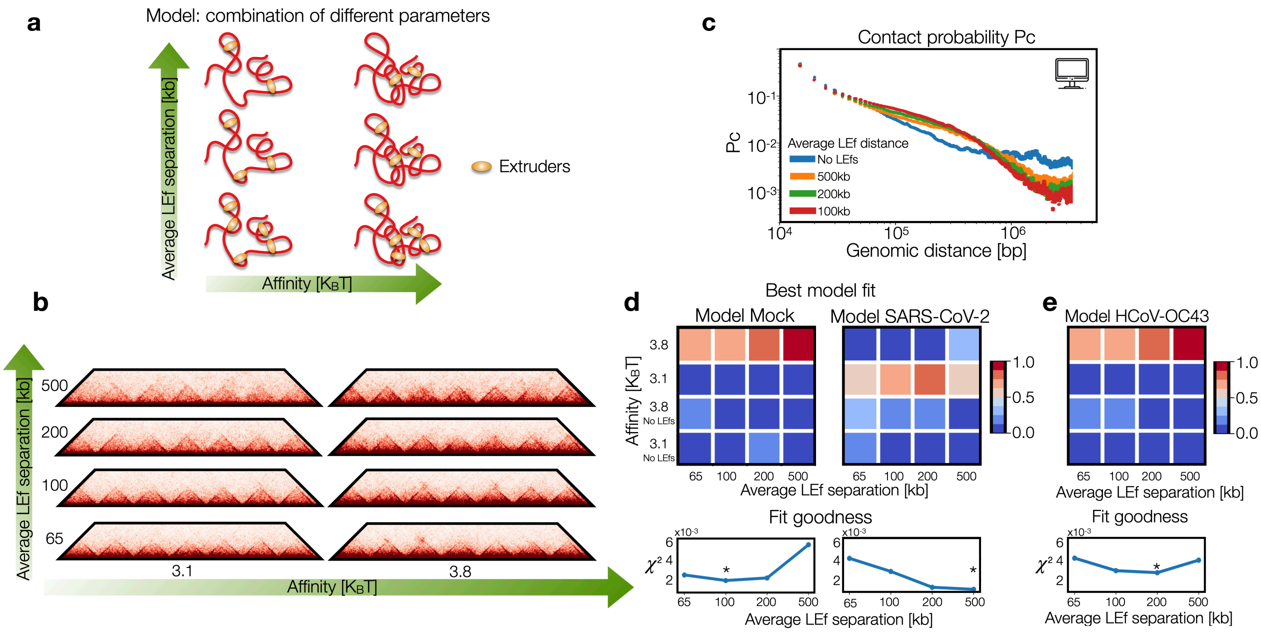

Supplementary Fig. 6: Viral infection impacts loop-extrusion and phase-separation features at TAD level. a The control parameters of the model are average separation between extruders (i.e. their number) and affinity between binders and polymer binding sites. Combination of models with different parameters are used to fit experimental HiC data. **b** Contact maps obtained from MD simulations for different parameters. **c** Contact probabilities for different average distances among extruders, affinity 3.1$K_B T$. **d** Best fit parameters obtained fitting experimental contact probabilities in Mock (left panel) and SARS-CoV-2 (right panel) conditions with a combination of probabilities fixing the average distance (i.e. a column) and varying binding affinities (first and second rows). The fit includes also the curves with no extruders (third and fourth rows). Each pixel is the normalized coefficient of the best linear combination returned by the fitting procedure (Methods). Below the χ^2 of each fit is shown. **e** As panel d, the fit performed on contact probabilities from HiC data in cells infected with HCoV-OC43[1]. Source data are provided as a Source Data file.

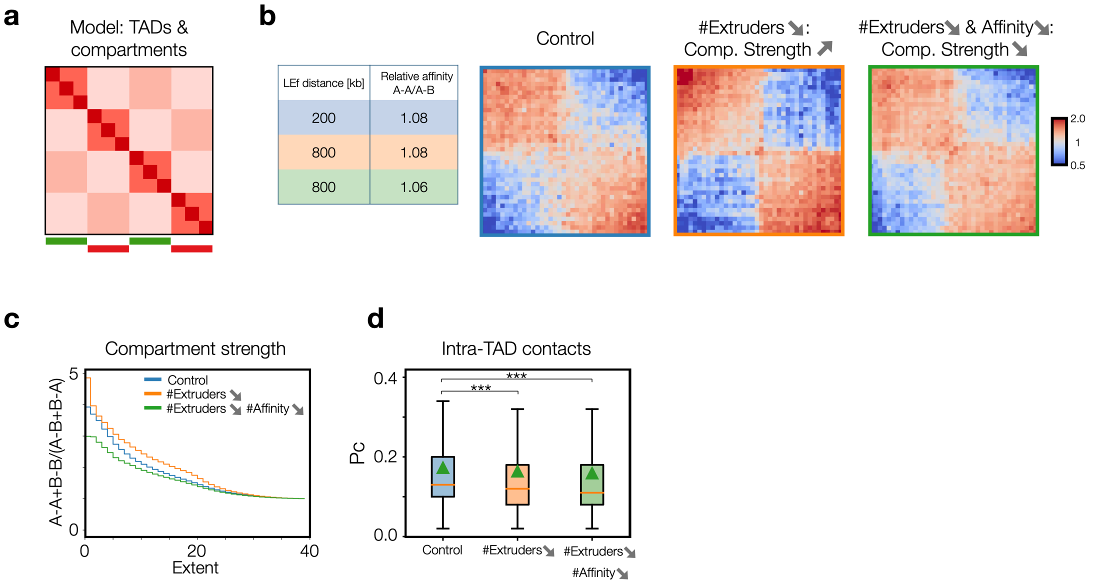

Supplementary Fig. 7: Combined alteration of loop-extrusion and phase-separation explains compartment weakening in SARS-CoV-2 infected cells. **a** Polymer model includes either loop-extrusion, driving TADs formation, and phase-separation affinities, driving compartment formation. **b** Saddle-plots corresponding to different system parameters (distance between extruders or, equivalently their number, and affinity). Reduction of extruders only leads to compartment strengthening. Combined reduction of extruders and affinity leads to compartment weakening. **c** Compartment strength profile obtained for different model parameters. **d** Distribution of intra-TAD contact probabilities (Pc) obtained for different model parameters. From left to right n=15298, n=15300, n=15296. The centre lines represent medians; triangles represent averages; box limits indicate the 25th and 75th percentiles; and whiskers extend 1.5 times the IQR from the 25th and 75th percentiles. Source data are provided as a Source Data file.

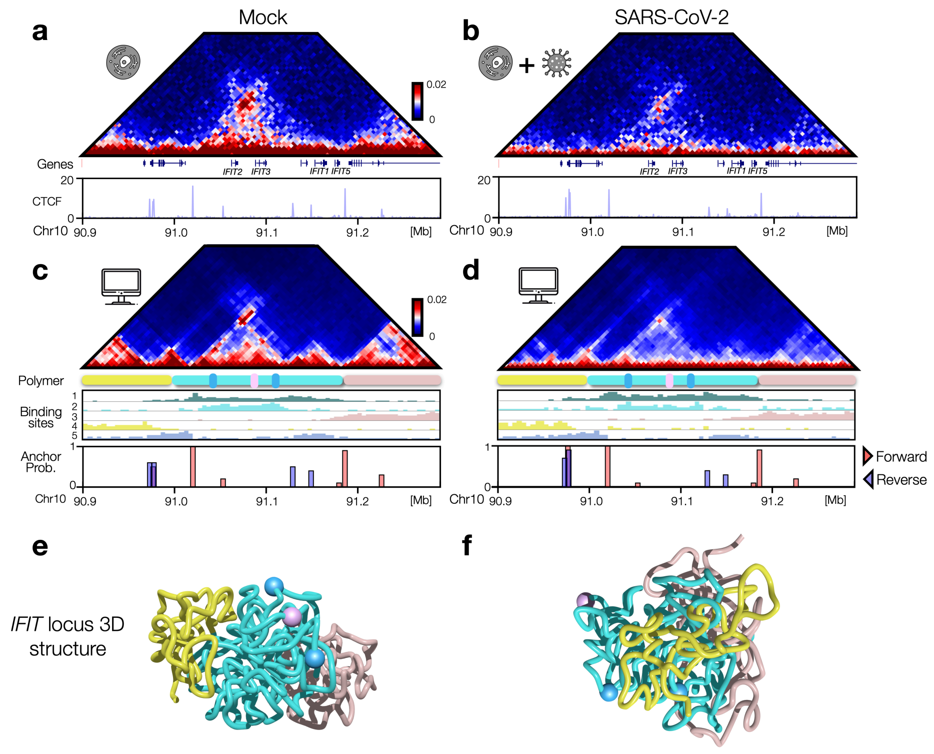

Supplementary Fig. 8: Structural re-arrangements of IFN *IFIT* locus. **a** HiC data of genomic region chr10:90900000-91290000 centered around the interferon response *IFIT* gene cluster, in Mock condition. Below, CTCF signal is shown (data taken from Ref.[1]). **b** As panel a, HiC and CTCF data in SARS-CoV-2 infected condition. **c** Contact map from MD simulations of the polymer model in Mock condition. Below, binding sites profile, anchor point probability and their orientation (Methods) is shown. **d** As panel c, model for SARS-CoV-2 infected condition. **e** Example of 3D structure of *IFIT* locus taken from an MD simulation of the Mock model. Different regions are differently colored according to the pattern of the contact maps. Pink and cyan spheres highlight the position of *IFIT* and its enhancers respectively. **f** As panel e, model for SARS-CoV-2. Source data are provided as a Source Data file.

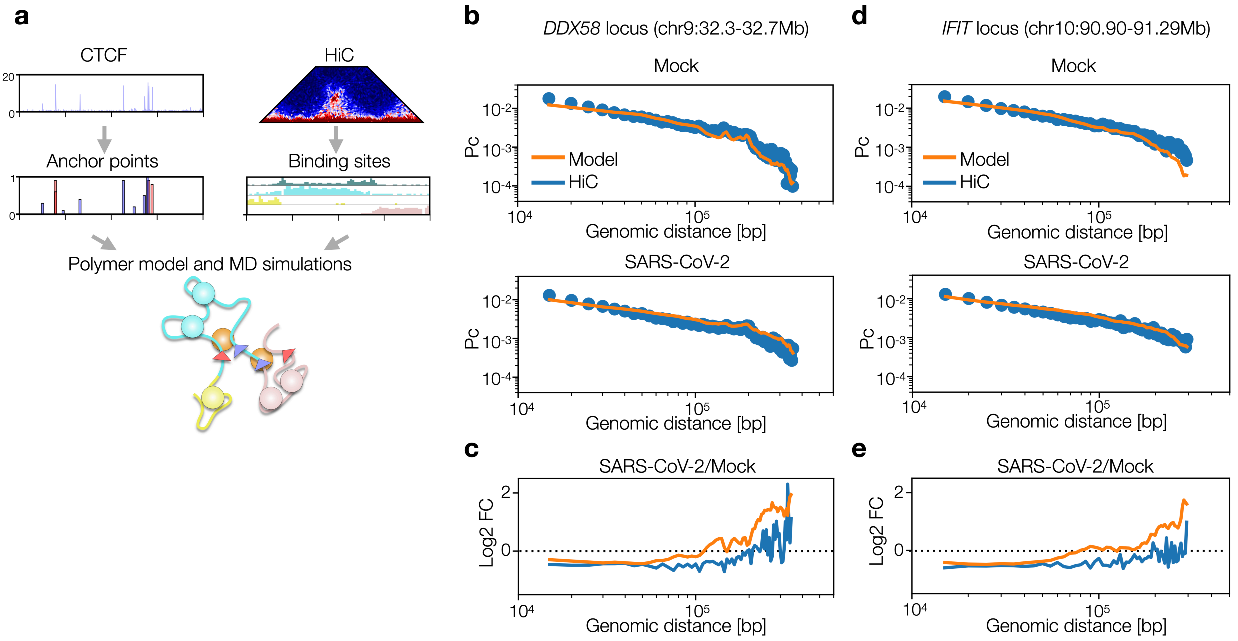

Supplementary Fig. 9: Polymer models of genomic loci with IFN genes. **a** Polymer model that simulates real loci include loop-extrusion and chromatin phase-separation driven by interaction with binders. Anchor points and probability (Methods) are obtained from CTCF data[2,3] and standard motif finding (FIMO) analysis. Binding sites are inferred from HiC data through the PRISMR method[4]. **b** Experimental (blue curve) and simulated (orange curve) contact probabilities of *DDX58* locus (shown in Fig. 3) in Mock (top panel) and SARS-CoV-2 infected (bottom panel) condition. **c** Log Fold Change SARS-CoV-2/Mock of experimental (blue) and model (orange) contact probabilities. Pearson correlation between the model and experimental data is r=0.81. **d** As panel b, for *IFIT* locus (shown in Supplementary Fig. 8). **e** As panel c, for *IFIT* locus. Pearson correlation between model and experimental data is r=0.66. Source data are provided as a Source Data file.

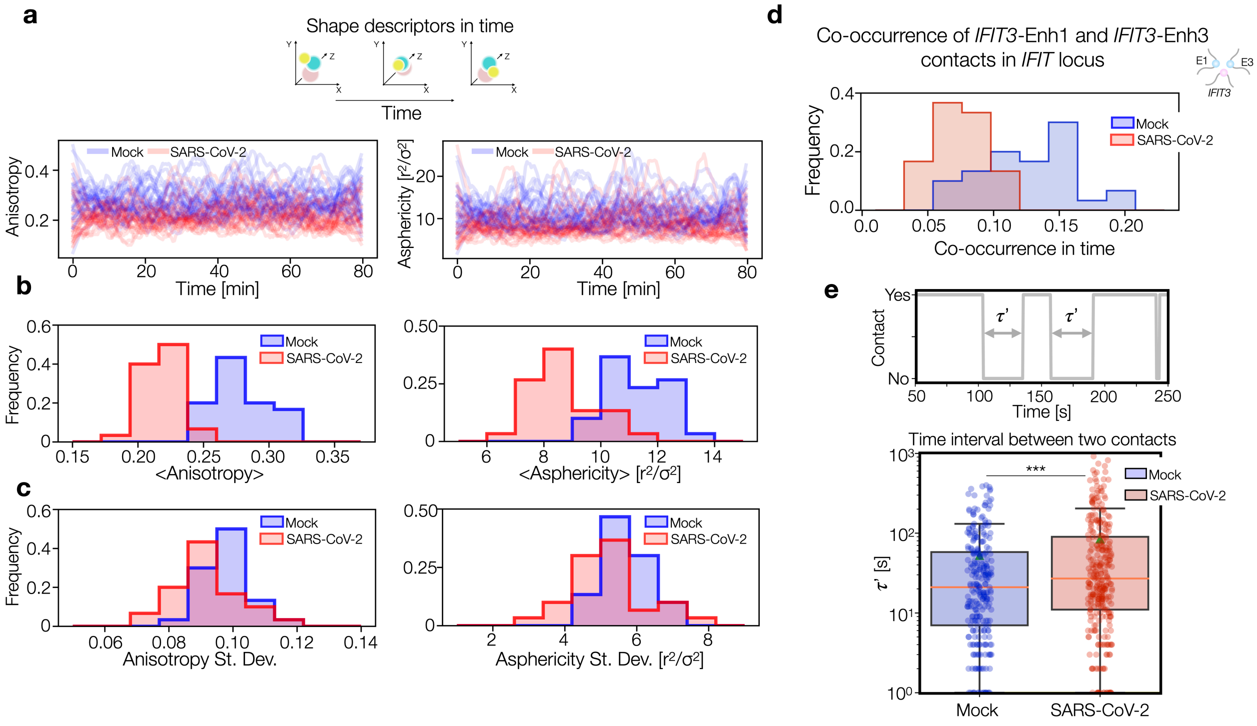

Supplementary Fig. 10: Time dynamics of shape descriptors and multiple contacts. a Evolution of 3D structure during time. Trajectories of polymer shape descriptors (Methods) anisotropy (left) and asphericity (right) in Mock (blue curves) and SARS-CoV-2 (red) models of *DDX58* locus. **b-c** Distributions of average distance (b) and standard deviation (c) computed over the independent time trajectories shown in panel a. Mock model tend to be more anisotropic and aspherical, standard deviations exhibit more overlapping distributions. n=30 independent trajectories for each model. **d** Distribution of fraction of co-occurrences at *IFIT* locus, i.e. how frequently *IFIT3*-E1 and *IFIT3*-E3 contacts are simultaneously detected during a trajectory (Methods). **e** Boxplots showing the distribution of time intervals τ' between consecutive contacts in Mock (blue) and SARS-CoV-2 (red) models. Distributions have statistically different averages (p-val=$3*10^{-4}$, one-sided t-test). n=267 for Mock and n=308 for SARS-CoV-2 infected model. The centre lines represent medians; triangles represent averages; box limits indicate the 25th and 75th percentiles; and whiskers extend 1.5 times the IQR from the 25th and 75th percentiles. Source data are provided as a Source Data file.

a

b

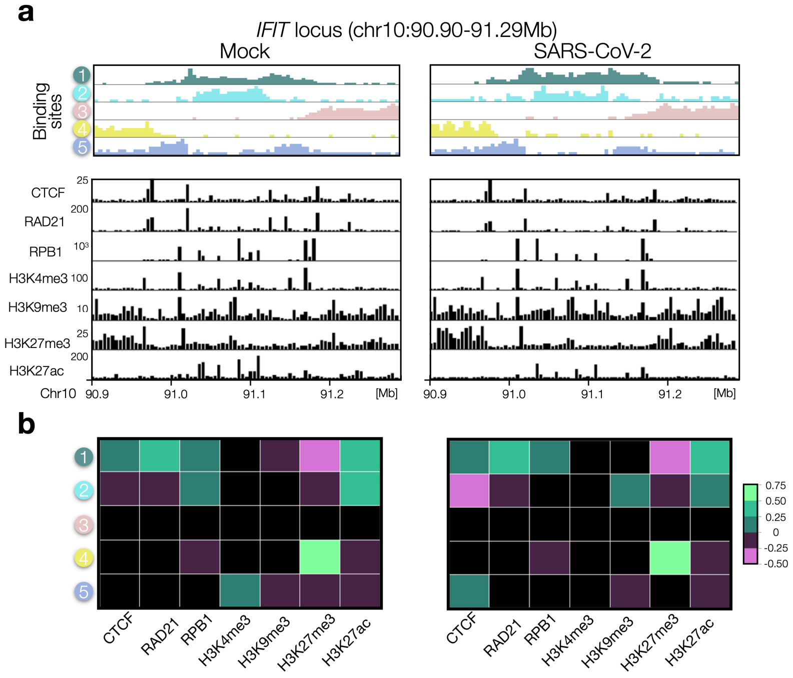

Supplementary Fig. 11: Chromatin re-arrangements correlates with a combination of changes of CTCF and histone marks at *IFIT* locus. a Top panel: distribution of binding sites obtained from HiC data in Mock (left) and SARS-CoV-2 (right) conditions. Bottom panel: different epigenetic marks (data from Ref.[1]). In SARS-CoV-2 relevant reductions of RAD21 and H3K27ac are observed. **b** Cross-correlation analysis between binding site profiles and epigenetic marks. Significative correlations (Methods) at *IFIT* locus in Mock (left) and SARS-CoV-2 (right) models are shown. Source data are provided as a Source Data file.

References

1. R. Wang, *et al.*, SARS-CoV-2 restructures host chromatin architecture. *Nat. Microbiol. 2023 84* **8**, 679-694 (2023).

2. A. L. Sanborn, *et al.*, Chromatin extrusion explains key features of loop and domain formation in wild-type and engineered genomes. *Proc. Natl. Acad. Sci.* **112**, E6456-E6465 (2015).

3. G. Fudenberg, *et al.*, Formation of Chromosomal Domains by Loop Extrusion. *Cell Rep.* **15**, 2038-2049 (2016).

4. S. Bianco, *et al.*, Polymer physics predicts the effects of structural variants on chromatin architecture. *Nat. Genet.* **50**, 662-667 (2018).
