# Supplementary material for: Multiscale modelling of chromatin 4D organization in SARS-CoV-2 infected cells
Source: Nat Commun. 2024 May 13;15:4014. doi: 10.1038/s41467-024-48370-6 (PMC11091192; doi:10.1038/s41467-024-48370-6)
Supplement: Supplementary file 3 — Description of Additional Supplementary Files [file 41467_2024_48370_MOESM3_ESM.pdf]

Description of Additional Supplementary Files

Supplementary Data 1: 3D coordinates of polymer structures of DDX58 locus.

Supplementary Movie 1: Polymer structure representing TADs in Mock condition, obtained from MD simulations of Mock model. For visualization purposes, only the three central TADs are shown.

Supplementary Movie 2: Polymer structure representing TADs in SARS-CoV-2 infected condition, obtained from MD simulations of SARSCoV-2 model. For visualization purposes, only the three central TADs are shown.

Supplementary Movie 3: Time dynamics of 3D structure in DDX58 locus, Mock condition, obtained from MD simulations. Below, genomic coordinates and color scheme are reported.

Supplementary Movie 4: Time dynamics of 3D structure in DDX58 locus, SARS-CoV-2 infected condition, obtained from MD simulations. Below, genomic coordinates and color scheme are reported.

Supplementary Software 1 : Scripts and commands to run Molecular Dynamics simulations of A/B compartments, TADs and DDX58 locus.
