# Supplementary material for: Multiscale modelling of chromatin 4D organization in SARS-CoV-2 infected cells
Source: Nat Commun. 2024 May 13;15:4014. doi: 10.1038/s41467-024-48370-6 (PMC11091192; doi:10.1038/s41467-024-48370-6)
Supplement: Supplementary file 5 — Supplementary Software 1 [file 41467_2024_48370_MOESM5_ESM.zip › Supplementary_Software_1/simulation_notebook.docx]

**Documentation of Molecular Dynamics polymer simulation and output analysis**

**Installation of the software and scripts**

The Molecular Dynamics (MD) simulation of the loci that are described in the article is implemented in python using a general-purpose particle simulation toolkit known as HOOMD-Blue (<https://glotzerlab.engin.umich.edu/hoomd-blue/>). The scripts used in the study are publicly available in the GitHub repository <https://github.com/ehsanirani/PhaseSeparation-LoopExtrusion-MD> and simulate a polymer model including loop extrusion and phase-separation mechanisms.

In order to test out the model presented in the article, the reader is invited to install the HOOMD-Blue python package and download the scripts from the GitHub repository. For the ease of use, we provide a Demo folder that includes these scripts, with the custom modifications that are required for the studies presented in the article. Please note that the scripts are written in HOOMD-Blue version 2.9x, which also requires a python version 3.8x or below. It is also necessary to install the gsd python package since the HOOMD-Blue saves its simulation data in the gsd file format. Other dependencies and prerequisites are mentioned on the installation page of the HOOMD-Blue website.

**Scripts and the input files**

The GitHub folder contains the main script for the MD simulation sbs-le.py and its three dependent scripts, saw.py, sbs_class.py and le4hoomd.py. It also has the cont-dist-map-sbs-le.py and cont-dist-map-sbs-le2HDF.py scripts, which produce the polymer contact maps and median distance maps from the simulation outputs, in .txt and HDF file formats, respectively.

The main script sbs-le.py starts out by defining the simulation context. It sets up the simulation environment and through the saw.py script, it prepares an initial self-avoiding walk polymer. The sbs_class.py is then used to create the context for the Strings-Binders-Switch (SBS) model on the polymer. The le4hoomd.py script augments the loop extrusion (LE) mechanism onto this SBS polymer, which includes the loop extrusion agents and the anchors. The sbs-le.py then defines the SBS-specific and average bead-binder interactions as well as the excluded volume particle interactions, and finally, the main simulation run and the periodic sampling of the data. These scripts are general purpose and they can be used to model any SBS model, with or without the loop extrusion mechanism incorporated into it.

Three input files are required to perform a full model simulation and they are specific to the particular model that we are interested in. First, the SBS polymer file indicates the structure of the polymer to be used. It has to be encoded in the .bed file format, where each line of the file corresponds to the coarse-grained beads of the SBS polymer and specifies the space-separated list of the type of the sub-beads contained in each bead. This file, thus implicitly defines the length of the polymer, the SBS domains as well as its resolution. Next, the loop extrusion anchor file specifies the probability of finding a backward or a forward anchor for each sub-bead of the polymer. These values would be used by the main script to probabilistically setup the anchors on the polymer. Finally, the settings file specifies the simulation context (simulation time steps, simulation box size etc.), the SBS bead-binder interaction parameters, loop extrusion parameters etc. The paths to the SBS polymer and the loop-extrusion anchor files are also needed to be specified in the settings file. All these files have to be present inside the same folder as the main script, either as standalone files or inside a subfolder.

**Simulation run**

To make a simulation run, use the command:

python3 sbs-le.py --settings $SETTINGS_FILE --sim-id demo0 --hoomd cpu

where $SETTINGS_FILE is the path to the settings file. In the Demo folder, we provide settings file for polymer model of DDX58 locus (settings_sbs_le_Mock.py and settings_sbs_le_SARS-CoV-2.py) as well as the polymer.bed and CTCF.bed files, required to completely define the simulation setup. Analogous files are provided for the A/B compartment and for the TAD polymer model. The --sim-id flag is used to specify the name of the simulation. If the system has a GPU available, a string gpu can be passed into the --hoomd flag instead of cpu to avail it. This would obviously facilitate a much faster simulation of the model.

**Expected output and the analysis**

At the completion of the simulation run, three output files – demo0-dump.gsd, dem0-restart.gsd and demo0-dump-ctcf.txt - are produced. The main output file demo0-dump.gsd has the snapshots of the simulated system saved at periodic intervals and it would be used to conduct the analysis of the simulation. The demo0-dump-ctcf.txt file has the loop extrusion anchors that were active for this simulation run, according to the probabilities specified in the loop extrusion anchor file. The dem0-restart.gsd file can be used to restart the simulation from the last timestep, if required.

The output file can be read using the gsd python package and then analyzed using any standard python scientific or numeric packages. For more details on the system snapshot information written by HOOMD-Blue into a gsd file, please refer to the gsd.hoomd documentation <https://gsd.readthedocs.io/en/v3.2.1/python-module-gsd.hoomd.html>. As an example, in the Demo folder we provide the cont-dist-map-sbs-le.py script. To run the script, use the command:

python3 cont-dist-map-sbs-le.py -i demo0-dump.gsd -o heatmap --particles $P --t1 0.7 --t2 1.0 --step 0.003 --contact-thr 3.0 --dist TRUE

$P specifies the total length of the model polymer (for example, in DDX58 locus model $P = 900). The output is the contact map of the simulated polymer in matrix format, averaged over the sampled system snapshots from 0.7^th^ fraction of the total timesteps till the end of the simulation, with a contact threshold of ~~5~~3σ (σ being the MD unit length). If the flag --dist has been set to TRUE then the corresponding median distance map would also be produced.
