# Supplementary material for: Multiscale modelling of chromatin 4D organization in SARS-CoV-2 infected cells
Source: Nat Commun. 2024 May 13;15:4014. doi: 10.1038/s41467-024-48370-6 (PMC11091192; doi:10.1038/s41467-024-48370-6)
Supplement: Supplementary file 10 — Reporting Summary [file 41467_2024_48370_MOESM10_ESM.pdf]

nature portfolio

Corresponding author(s): Andrea Maria Chiariello

Last updated by author(s): Apr 15, 2024

Reporting Summary

Nature Portfolio wishes to improve the reproducibility of the work that we publish. This form provides structure for consistency and transparency in reporting. For further information on Nature Portfolio policies, see our Editorial Policies and the Editorial Policy Checklist.

Statistics

For all statistical analyses, confirm that the following items are present in the figure legend, table legend, main text, or Methods section.

n/a	Confirmed	
☐	☒	The exact sample size (*n*) for each experimental group/condition, given as a discrete number and unit of measurement
☐	☒	A statement on whether measurements were taken from distinct samples or whether the same sample was measured repeatedly
☐	☒	The statistical test(s) used AND whether they are one- or two-sided<br>*Only common tests should be described solely by name; describe more complex techniques in the Methods section.*
☒	☐	A description of all covariates tested
☒	☐	A description of any assumptions or corrections, such as tests of normality and adjustment for multiple comparisons
☐	☒	A full description of the statistical parameters including central tendency (e.g. means) or other basic estimates (e.g. regression coefficient) AND variation (e.g. standard deviation) or associated estimates of uncertainty (e.g. confidence intervals)
☐	☒	For null hypothesis testing, the test statistic (e.g. *F*, *t*, *r*) with confidence intervals, effect sizes, degrees of freedom and *P* value noted<br>*Give P values as exact values whenever suitable.*
☒	☐	For Bayesian analysis, information on the choice of priors and Markov chain Monte Carlo settings
☒	☐	For hierarchical and complex designs, identification of the appropriate level for tests and full reporting of outcomes
☐	☒	Estimates of effect sizes (e.g. Cohen's *d*, Pearson's *r*), indicating how they were calculated

Our web collection on statistics for biologists contains articles on many of the points above.

Software and code

Policy information about availability of computer code

Data collection	No software was used
Data analysis	We used the publicly available HOOMD-Blue software (version 2.9.6) for the Molecular Dynamics simulations of the polymer models. Analyses involving polymer spatial coordinates were performed using buit-in functions within the Python Scipy package (version 1.6.2). Positions and orientations of the model anchor sites were determined by standard motif finding analysis using the FIMO tool within the MEME Suite software (version 5.5.3). Analysis of HiC data and simulated contact maps were performed using the open source Cooler software and the cooltools package (https://github.com/open2c/cooler). All codes required to perform the simulations of the polymer models are provided at the publicly available repository: https://github.com/ehsanirani/PhaseSeparation-LoopExtrusion-MD.

For manuscripts utilizing custom algorithms or software that are central to the research but not yet described in published literature, software must be made available to editors and reviewers. We strongly encourage code deposition in a community repository (e.g. GitHub). See the Nature Portfolio guidelines for submitting code & software for further information.

Data

Policy information about availability of data

All manuscripts must include a data availability statement. This statement should provide the following information, where applicable:

- Accession codes, unique identifiers, or web links for publicly available datasets
- A description of any restrictions on data availability
- For clinical datasets or third party data, please ensure that the statement adheres to our policy

Published HiC data and ChIP-seq data used in this work are available at the Gene Expression Omnibus (GEO) database with accession number GSE179184. CTCF binding motif is available from the JASPAR database (matrix profile MA0139.1). Polymer configurations of DDX58 locus are provided as Supplementary Data 1. The polymer structures generated for the IFIT locus and Chr11 will be available from the authors upon request. Please contact Andrea M. Chiariello at andreamaria.chiariello@infn.it. Requests of these data will be answered within approximately two weeks. Source data are provided with this paper.

Research involving human participants, their data, or biological material

Policy information about studies with human participants or human data. See also policy information about sex, gender (identity/presentation), and sexual orientation and race, ethnicity and racism.

Reporting on sex and gender	Not applicable to this study
Reporting on race, ethnicity, or other socially relevant groupings	Not applicable to this study
Population characteristics	Not applicable to this study
Recruitment	Not applicable to this study
Ethics oversight	Not applicable to this study

Note that full information on the approval of the study protocol must also be provided in the manuscript.

Field-specific reporting

Please select the one below that is the best fit for your research. If you are not sure, read the appropriate sections before making your selection.

☒ Life sciences ☐ Behavioural & social sciences ☐ Ecological, evolutionary & environmental sciences

For a reference copy of the document with all sections, see nature.com/documents/nr-reporting-summary-flat.pdf

Life sciences study design

All studies must disclose on these points even when the disclosure is negative.

Sample size	Our study is based on computer simulations of polymer models. No statistical methods were used to predetermine sample sizes. For each of the studied model loci, we generated ensembles of at least 2000 conformations, ensuring robustness of statistical analyses.
Data exclusions	No data were excluded.
Replication	Our study is based on computer simulations and we do not generate new biological data. We verified the reproducibility of the findings of our manuscript running an additional bunch of 50 independent simulations for each studied locus.
Randomization	Our study is based on computer simulations of polymer physics models and compare it with re-analyzed publicly available genomic datasets. Randomization is not relevant to this study.
Blinding	Our study is based on computer simulations of polymer physics models and compare it with re-analyzed publicly available genomic datasets. Blinding is not relevant to this study.

Reporting for specific materials, systems and methods

We require information from authors about some types of materials, experimental systems and methods used in many studies. Here, indicate whether each material, system or method listed is relevant to your study. If you are not sure if a list item applies to your research, read the appropriate section before selecting a response.

Materials & experimental systems

n/a	Involved in the study
☒	☐ Antibodies
☒	☐ Eukaryotic cell lines
☒	☐ Palaeontology and archaeology
☒	☐ Animals and other organisms
☒	☐ Clinical data
☒	☐ Dual use research of concern
☒	☐ Plants

Methods

n/a	Involved in the study
☒	☐ ChIP-seq
☒	☐ Flow cytometry
☒	☐ MRI-based neuroimaging

Plants

Seed stocks

Report on the source of all seed stocks or other plant material used. If applicable, state the seed stock centre and catalogue number. If plant specimens were collected from the field, describe the collection location, date and sampling procedures.

Novel plant genotypes

Describe the methods by which all novel plant genotypes were produced. This includes those generated by transgenic approaches, gene editing, chemical/radiation-based mutagenesis and hybridization. For transgenic lines, describe the transformation method, the number of independent lines analyzed and the generation upon which experiments were performed. For gene-edited lines, describe the editor used, the endogenous sequence targeted for editing, the targeting guide RNA sequence (if applicable) and how the editor was applied.

Authentication

Describe any authentication procedures for each seed stock used or novel genotype generated. Describe any experiments used to assess the effect of a mutation and, where applicable, how potential secondary effects (e.g. second site T-DNA insertions, mosiacism, off-target gene editing) were examined.
